# HIV prevalence and associated factors among married women, Mozambique, 2015: analysis of the 2015 National AIDS Indicator Survey (IMASIDA)

**DOI:** 10.11604/pamj.2024.47.94.42781

**Published:** 2024-02-28

**Authors:** Samuel Nuvunga, Denise Chitsondzo Langa, Jahit Sacarlal, Erika Rossetto, Cynthia Semá Baltazar

**Affiliations:** 1 Mozambique Field Epidemiology Training Program, National Institute of Health, Maputo, Mozambique,; 2 National Institute of Health, Maputo, Mozambique,; 3 Faculty of Medicine, Eduardo Mondlane University, Maputo, Mozambique,; 4 MassGenics, assigned to Mozambique Centers for Disease Control Prevention, Maputo, Mozambique

**Keywords:** HIV infections, prevalence, risk factors, women, Mozambique

## Abstract

**Introduction:**

epidemiological estimates from the 2021 Joint United Nations Program on HIV/AIDS (UNAIDS) emphasize the existing gender disparities, where women face a higher risk of HIV/AIDS exposure. In Mozambique, as of 2021, the HIV prevalence rate among the adult population stood at 12.5%, with an even more concerning rate of 15.4% among women of reproductive age.

**Methods:**

a cross-sectional study was carried out with secondary data from the Survey on National Indicators of Vaccination, Malaria, and HIV/AIDS (IMASIDA 2015), where we included married women, both civil marriage and common law marriage of reproductive age 15-49 years. Statistical analyses, including chi-squared tests and logistic regression models, accounting for survey design, were employed to assess associations.

**Results:**

the study findings showed that HIV prevalence was higher among married women aged 35-49 years (aOR=2.5; 95% CI: 1.3-4.6; p=0.005), those without formal education (aOR=7.7; 95% CI: 1.1-52.9; p=0.038) and those with primary education (aOR=9.8; 95% CI: 1.6-60.1; p=0.014), those who experienced domestic violence (aOR=1.8; 95% CI: 1.0-3.2; p=0.04), had an uncircumcised partner (aOR=1.9; 95% CI: 1.2-3.1; p=0.008), and had three or more lifetime sex partners (aOR=3.6; 95% CI: 2.9-7.3; p<0.001). Women who were in one lifelong union had a lower risk of HIV positivity (aOR=0.5; 96%CI: 0.3-0.8, p=0.005).

**Conclusion:**

the findings of this study highlight sociodemographic, behavioral, and violent factors associated with HIV prevalence among women. These findings underscore the importance of targeted interventions and education programs aimed at reducing HIV transmission among females and promoting safer sexual practices.

## Introduction

Human immunodeficiency virus (HIV) continues to be a significant global public health challenge, with far-reaching socio-economic implications, especially in developing countries. According to the 2022 Joint United Nations Program on HIV/AIDS (UNAIDS) data, continues to be a significant global public health challenge, with far-reaching socio-economic implications 38.4 million people worldwide were living with HIV, with 1.5 million new HIV infections in 2021 [1]. Notably, a substantial portion of these new infections occurred among women and girls, accounting for 46% of all new HIV cases globally in 2022. The 2021 UNAIDS epidemiological estimates underscored the persistent gender disparities in HIV prevalence, placing women at elevated risk of HIV exposure, particularly in sub-Saharan Africa [2].

Mozambique, a nation grappling with one of the most severe HIV burdens globally, highlights the urgent need for targeted interventions. According to the latest Mozambique Population-based HIV Impact Assessment (PHIA) data from The Mozambique Population-Based HIV Impact Assessment 2021 (INSIDA) 2021, the HIV prevalence among individuals aged 15-45 years stands at 12.4%, with a notably higher prevalence among females at 15.4% compared to males at 9.0% [3]. The pattern of new infections initially increased until 2012, followed by a decrease until 2022. During this period, approximately 54,000 new infections occurred among women, and 30,000 cases were reported among men [4].

The relationship between HIV and married women is intricately shaped by a web of socio-cultural, economic, and behavioral factors. Married women can be vulnerable to HIV transmission from their spouses, especially if there is a lack of knowledge or resources to negotiate safe sexual practices. Gender disparities further exacerbate the risk, as women often contend with reduced decision-making power regarding sexual activity and condom use within marriages [5-7].

Despite the growing number of HIV infections in the general population, particularly among women, there is a paucity of research specifically focused on married women in Mozambique. It is important to highlight that married women may underestimate their risk of HIV acquisition, either presuming their partners' monogamy or due to challenges in negotiating safe sexual practices, often rooted in fears of violence or the potential dissolution of the marriage. This cross-sectional study was conducted with the objective of estimating the prevalence of HIV/AIDS and analyzing the associated risk factors among married women of reproductive age in Mozambique using data from the Mozambique AIDS Indicator Survey 2015.

## Methods

**Study design:** a cross-sectional study was carried out with secondary data from the Survey on National Indicators of Vaccination, Malaria, and HIV/AIDS (IMASIDA 2015). to estimate the prevalence and analyze factors associated with HIV/AIDS among married women of reproductive age in Mozambique.

**Study setting and population:** Mozambique is a country on the eastern coast of Southern Africa with an area of 799,380 km^2^, its limits are: to the north, Tanzania; to the northwest, Malawi, and Zambia; to the west, Zimbabwe, South Africa and Swaziland; to the south, South Africa; to the east, the section of the Indian Ocean known as the Mozambique Channel. The country is divided into 11 provinces and 144 districts, with each province having a provincial governor and a district administration in each district [8].

This study utilized data the 2015 HIV/AIDS Indicator Survey in Mozambique - The National Indicators of Immunization, Malaria and HIV/AIDS Survey (IMASIDA 2015). The IMASIDA survey was cross-sectional and carried out in two phases with the application of stratification methods and cluster sampling to ensure that, for each province, inferences were possible with almost the same precision. Enumeration Areas (AEs), households, and individuals were primary sampling units, secondary sampling units, and tertiary sampling units, respectively.

The IMASIDA survey randomly selected 307 AEs from 45,000 AEs defined according to the 2007 General Population and Housing Census, 134 AEs from urban areas, and 173 AEs from rural areas. A fixed number of households were systematically selected in each AE. Men and women aged between 15 and 59 years participated in individual face-to-face interviews and provided a blood sample for HIV testing. Participants who declared being HIV-positive were not tested for HIV. The dataset for this survey can be accessed at the Demographic and Health Surveys (DHS) Program [9].

Due to the unproportionate assignment of samples by districts and differentials in response rates, the sampling weighting of the results of IMASIDA 2015 should be used in all analyses to ensure nationally representative results. For our study, the data were calculated using the same weights applied to the IMASIDA results. For more details about sample probabilities and sampling weights visit the IMASIDA report [10].

**Variables:** the variables included in our study are, age group (years), main language spoken, religion, education level, employment, area of residence, zone of residence, quintile of wealth, number of unions throughout life, age of first cohabitation, husband/partner circumcised, had any sexually transmitted infection (STI) in last 12 months, total lifetime number of sex partner, consistent condom use with most recent partner in the last 12 months and domestic violence. The outcome variable was a positive HIV result. We considered possible confounders in our analysis.

### Data resource and measurement

**Data collection tool:** in IMASIDA, four questionnaires were used: one to interview households, an individual questionnaire for women aged 15-59, an individual questionnaire for men aged 15-59 and another for biometrics for men and women aged 15-59.

**Data collection:** field work for data collection was carried out by 25 teams, including central technicians in the first phase. Of the eleven provinces, eight had two teams and three had three teams, depending on the size of the population and the prevalence of HIV and AIDS. Each province had a supervisor responsible for ensuring survey quality. Each team consisted of a controller, an enumerator, two enumerators, a field typist, and two health technicians (counselors) responsible for the testing process, blood sample collection, referral, and treatment of malaria. The data collection activity was preceded by the social mobilization activity in the 307 enumeration areas (AE) selected for IMASIDA 2015. During the data collection activities, several levels of quality control were applied. Samples of approximately 1 ml of blood were collected in adults and 0.5 ml in children, through a finger prick in adults and children over 18 months of age and a heel prick in malnourished children under 18 months and/or thin. For each participant who agreed to be tested for HIV, the Determine HIV-1/2 test was initially performed. Non-reactive samples in the screening test were classified as HIV-negative. For samples whose screening test was reactive, a confirmatory test was carried out. Reactive samples in both tests were classified as HIV positive.

**Sample size:** first, the criteria for selecting secondary data for the study was defined as a database of women only, and then data from married women and women in marital unions of reproductive age (15-49 years) were selected, and presented results data of HIV testing at a national level and we excluded data from all participants who were married and in a marital union under the age of 15 and over 49 years of age, with omitted data whose HIV test result was indeterminate. The primary database of IMASIDA was composed of 7,749 women aged between 15 and 59 years old, of which 2,823 were married. we restricted our population sample to include only married women of reproductive age (15-49 years). The final unweighted sample consisted of 2,669 women and the final weighted sample was calculated as 3,006 women.

**Data analysis:** data were analyzed using STATA software version 16.1. Descriptive analyses were conducted to summarize socio-demographic and behavioral characteristics using frequencies, percentages, averages, and standard deviations. Pearson's chi-square tests were used in bivariate analyses to verify the association between categorical variables.

The primary independent variable in this analysis reflects the current marital status of women as reported during the implementation of the survey. The available response categories for this variable include “never married”, “married or in a common-law marriage”, “divorced”, “separated”, or “widowed”.

In a first phase, a bivariate analysis of each factor in association with HIV was performed. Factors that presented evidence of association (p<0.20) with positive HIV test results in the bivariate analysis were considered a hypothesis of association and included in the multivariate analysis to obtain the association of interest and in the end, a multivariate logistic regression was used to estimate adjusted odds ratios. A 95% confidence interval and a significance level of p<0.05 were used to define statistical significance in the final model, noting that all analyzes were adjusted to take into account the complex sample design.

**Ethical considerations:** the final databases of the Democratic Health Survey (DHS) program surveys, which are publicly available on the DHS program [9], adhere to strict ethical standards and ensure participant confidentiality. The data are entirely anonymous. The IMASIDA survey conducted in 2015 adhered to both Mozambican and international ethical guidelines and was approved from the Mozambique National Committee for Bioethics in Health (CNBS) under the code IRB0002657, with reference 262/CNBS/14. The IMASIDA participants provided free and informed consent before participating in the study.

Given that this study is a secondary analysis, the database provided by the National Institute of Health (NIH) to interested researchers is likewise, therefore maintains strict ethical standards.

## Results

**Sociodemographic characteristics and self-reported domestic violence:** our analysis consisted of 3,006 married women, where 59% (1,777/3,006) were officially married and 41% (1,229/3,006) were in common-law marriages. The mean age was 30 years (STD=8.9), and 35% (1,060/3,006) were in the age group of 15 to 24 years. The majority, approximately 85% (2,546/3,006), spoke a local tribal language as their native tongue, while 6% (175/3,006) identified Portuguese as their native language. Most women were Catholic (32%; 975/3,006), followed by Protestant (16%; 478/3,006). The study found that 55% of women had an elementary education (1660/3,006) and that 32% of women had no formal education (946/3,006). Forty-six percent (1384/3,006) of women fell within the bottom two wealth quintiles. Approximately 7.5% of women in civil marriages and 6.5% of those in common-law marriages reported experiencing acts of domestic violence (Table 1).

**Table 1:** sociodemographic characteristics and self-reported domestic violence among married women in reproductive age, Mozambique, 2015

Characteristics	Civil marriage^1^	Common-law marriage^2^	Total
	(n = 1777)	(n = 1229)	(n = 3006)
	n (%)	n (%)	n (%)
**Age group (years)**			
15-24	615 (34.6)	445 (37.0)	1060 (35.3)
25-34	558 (31.4)	420 (34.2)	978 (32.5)
35-49	604 (33.9)	374 (30.4)	978 (32.5)
**Main language spoken**			
Portuguese	84 (4.7)	91 (7.4)	175 (5.8)
Mother tongue (native language)^3^	1528 (86.0)	1018 (82.8)	2546 (84.7)
Other	165 (9.3)	130 (10.6)	295 (9.8)
**Religion**			
Catholic	668 (37.6)	307 (24.9)	975 (32.4)
Protestant	249 (14.0)	232 (17.9)	481 (16.0)
Evangelical	112 (6.3)	188 (15.3)	300 (10.0)
Zione	100 (5.6)	203 (16.5)	303 (10.1)
Other	534 (30.1)	223 (18.1)	757(25.2)
None	114 (6.4)	86 (7.0)	200 (6.6)
**Education level**			
No education	618 (34.8)	328 (26.7)	946 (31.5)
Primary	944 (53.1)	716 (58.3)	1660 (55.2)
Secondary	194 (10.9)	185 (15.1)	379 (12.6)
Higher	21 (1.2)	10 (0.8)	31 (1.0)
**Employment**			
No	1164 (65.5)	684 (55.6)	1848 (61.5)
Yes	613 (34.5)	555 (45.2)	1168 (38.9)
**Area of residence**			
Urban	441 (24.8)	403 (32.8)	844 (28.1)
Rural	1336 (75.2)	836 (68.0)	2172 (72.3)
**Zone of residence**			
North	1031 (58.0)	372 (30.3)	1403 (46.7)
Center	650 (36.6)	411 (33.4)	1061 (35.3)
South	96 (5.4)	456 (37.1)	552(18.4)
**Quintile of wealth**			
Poorest	451 (25.4)	211 (17.1)	662 (22.0)
Poorer	498 (28.0)	224 (18.2)	722 (24.0)
Middle	373 (21.0)	270 (21.9)	643 (21.4)
Richer	261 (14.7)	258 (21.0)	519 (17.3)
Richest	194 (10.9)	276 (22.5)	470 (15.6)
**Domestic violence^4^**			
No	1643(92.5)	1150(93.5)	2793(92.9)
Yes	133(7.5)	80(6.5)	213(7.1)

1 : legally recognized marriage between two people who have not purchased a marriage license or had their marriage solemnized by a ceremony; ^2^: marriage performed, recorded and recognized by a government official, performed by a religious body and recognized by the state, or it may be entirely secular; ^3^ the term “mother tongue” refers to a person's native language - that is, a language learned from birth. Also called a first language, dominant language, home language, and native tongue (although these terms are not necessarily synonymous); ^4^ the United Nations defines violence against women as “any act of gender-based violence that results or may result in physical, sexual or mental harm or suffering to women, including threats of such acts, coercion or arbitrary deprivation of liberty, whether in public or private life”

**Factors associated with HIV among married women of reproductive age:** HIV prevalence among married women of reproductive age, including those in both official and common-law marriages, was found to be 6.9% (95% CI: 5.7-8.4). The lowest prevalence (0.1%) was observed in women with higher education and with one lifetime sexual partner. The highest HIV prevalence (6.3%) was among women who reported having had an STI in the past 12 months (Table 2).

**Table 2:** multivariate analysis of factors associated with HIV infection among married women in reproductive age, Mozambique, 2015

Variable	HIV+ (n/N)		aOR (95% CI)	p-value
	N	%		
**General**	**122**	**6.9**		
**Age group**				
15-24	19	1.1	Ref.	
25-34	24	1.3	1.6 [0.8-3.1]	0.161
35-49	79	4.5	2.5 [1.3-4.6]	0.005
**Main language spoken**				
Portuguese	14	0.8	Ref.	
Mother tongue (native language)	92	5.3	1.3 [0.7- 2.5]	0.3
Other	14	0.8	1.8 [0.8- 4.2]	0.1
**Education level**				
None	18	1.0	7.7 [1.1-52.9]	0.038
Primary	84	4.7	9.8 [1.6-60.1]	0.014
Secondary	20	1.1	3.1 [0.5-19.4]	0.221
Higher	1	0.1	Ref.	
**Type of marriage**				
Civil marriage	43	2.4	0.6 [0.3-1.1]	0.106
Common-law marriage	79	4.5	Ref.	
**Religion**				
Catholic	26	1.4	1.1 [0.5-2.2]	0.783
Protestant	38	2.1	1.2 [0.6-2.5]	0.645
Zione	15	0.9	0.7 [0.3-1.6]	0.415
Evangelical/pentecostal	19	1.1	0.7 [0.3-1.5]	0.366
Other	15	0.9	0.8 [0.3-2.2]	0.690
None	8	0.5	Ref.	
**Residence area**				
Urban	71	4.0	1.4 [0.7-2.6]	0.350
Rural	51	2.9	Ref.	
**Residence zone**				
North	14	0.8	Ref.	
Center	39	2.2	2.2 [0.8-6.2]	0.135
South	69	3.9	1.8 [0.6-5.2]	0.283
**Quintile of wealth**				
Poorest	6	0.3	Ref.	
Poorer	8	0.5	0.5 [0.1-2.0]	0.312
Middle	13	0.7	1.0 [0.3-3.0]	0.956
Richer	46	2.6	2.0 [0.6-5.9]	0.223
Richest	49	2.8	1.6 [0.4-5.9]	0.504
**Number of unions throughout life**				
1	71	4.0	0.5 [0.3-0.8]	0.005
>1	51	2.9	Ref.	
**Age of first cohabitation**				
<18	53	3.0	Ref.	
≥18	69	3.9	1.3 [0.8-2.1]	0.352
**Husband/partner circumcised**				
No	55	3.1	1.9 [1.2-3.1]	0.008
Yes	67	3.8	Ref.	
**Had any STI in last 12 months**				
No	111	6.3	0.4 [0.2-1.1]	0.0779
Yes	11	0.6	Ref.	
**Total lifetime number of sex partners**				
1	29	1.6	Ref.	
2	40	2.3	1.7 [0.9-3.1]	0.065
**≥3**	53	3.0	3.6 [1.9-7.0]	<0.0001
**Consistent condom use with most recent partner in the last 12 months**				
No	96	5.5	Ref.	
Yes	26	1.5	1.7 [1.1-2.8]	0.02
**Domestic violence**				
No	105	6.0	Ref.	
Yes	17	0.9	1.8 [1.0-3.2]	0.04

STI: sexually transmitted infection; Ref: reference

**Multivariate analysis:** all factors that were significant in bivariate analysis were put in multivariate analysis and only two were found to be statistically associated with the prevalence of HIV. Our analysis indicated that married women aged 35 to 49 years were more likely to be infected with HIV compared to those aged 15 to 24 years (aOR=2.5, 95%CI: 1.3-4.6, p=0.005). The odds of being HIV-positive were significantly higher among married women with no education (aOR=7.7, 95% CI: 1.1-52.9, p=0.038) and among women with elementary education (aOR=9.8, 95%CI: 1.6-60.1, p=0.014) versus those with higher education.

The results also showed that women who reported having only one union throughout their lives were 50% less likely to be HIV positive than those who reported more than one union throughout their lives (aOR=0.5, 95%CI: 0.3-0.8, p=0.005). Women who did not use condoms with their most recent partner in the past 12 months were more likely to be HIV-positive compared to those who used condoms (aOR=1.8, 95%CI: 1.1-2.8, p<0.0001). Additionally, compared with women who reported having a circumcised partner, the odds of being HIV-positive were significantly higher in women who reported having an uncircumcised partner (aOR=1.9, 95%CI: 1.2-3.1, p=0.008).

Women who reported more than three sexual partners in life were more likely to have an HIV-positive status compared to those who reported having only one sexual partner (aOR=3.6, 95%CI: 1.9-7.0, p<0,0001). Women who reported acts of domestic violence were more likely to have an HIV-positive status compared to those who reported not having suffered acts of domestic violence (aOR=1.8, 95% CI: 1.0-3.2, p=0.04).

Our findings showed that the odds of seropositivity were 1.7 times higher (p=0.02) in women who reported having used a condom consistently in their last relationship in the 12 months prior to the survey compared to those who reported not using condoms (these odds increased to 2.9 in the multivariate model). Some sociodemographic and behavioral characteristics such as type of marriage, religion, area of residence, wealth quintile, age at first cohabitation, and having had a sexually transmitted infection in the last 12 months were statistically significant in the bivariate model, however in the final model they did not show statistically significant association with HIV infection in the study population (Table 2).

## Discussion

Our study revealed that among the population of married women in Mozambique, there was a presence of HIV prevalence, though at a lower rate (6.9%) when compared to the HIV prevalence in the general reproductive-age population, which was 12.4% [11,12].

In this study, HIV-positive serostatus was significantly associated with age; older married women were more likely to contract HIV than younger women, other studies have also [11,13,14], revealed an association between older age and greater likelihood of HIV, where the odds of being HIV-positive increase with increasing age. This association suggests that the risk of HIV increases with age, possibly due to older women being at the peak of their sexual activity and having accumulated more exposure to HIV over time.

Lack or low levels of formal education were associated with an increased odds of being HIV-positive in this study. This finding aligns with previous research conducted by Patra (2016), Ekholuenetale *et al*. (2020) and Mocumbi *et al*. (2017), which also show an association between low education levels and HIV risk [15-17]. The lack of education may contribute to less awareness about disease transmission, prevention, and control [18-21].

Married women who reported multiple marriages over their lifetimes exhibited a higher likelihood of being HIV-positive. This heightened risk may be associated with increased exposure to infection each time they change sexual partners. In stable relationships, there tends to be a reluctance to use condoms due to established trust and stability. This trust in each new partner may lead to unprotected intercourse without prior testing for sexually transmitted diseases, subsequently elevating the risk of HIV transmission for women with multiple marriages [22,23].

Married women who reported experiencing domestic violence were more likely to test HIV-positive compared to those who did not report such experiences. This heightened risk can be attributed to the vulnerability these women face within their marital relationships. Both before and after infection, they encounter various aspects of this vulnerability, shaped by cultural and gender norms, traditional practices, emotional dependence, conformity, permissiveness, and secrecy, which weaken their position and impede control over their bodies and sexuality. Consequently, they find themselves in unequal and abusive relationships, increasing their susceptibility to contracting sexually transmitted infections from their partners [24,25].

The observed association between HIV infection and the circumcision status of male partners in our study aligns with the well-established body of research demonstrating the protective effect of male circumcision against HIV transmission. Male circumcision has been shown to significantly reduce the risk of HIV acquisition among heterosexual men, primarily due to the lower susceptibility of circumcised men to HIV infection during sexual intercourse [26-29].

However, in our analysis we unexpectedly found that women who reported consistently using condoms were more likely to be HIV-positive. We hypothesize that one reason for this finding could be that many women who consistently used condoms already knew their HIV-positive status, and therefore opted to regularly use condoms as a way of protecting their partner. Dias *et al*. (2018) and Xavier's *et al*. (2020) studies on risk factors for HIV/AIDS in women who are married or in a stable partnership revealed that women who are married or in stable relationship feel inhibited from demanding that their partner use a condom, fearing that their partners may think that if they demand condom use it is because they are being unfaithful [11,23]. Expectations about adhering to traditional gender norms within relationships and difficulties within marital relationships can affect HIV infection susceptibility and the adoption of risk-free behaviors. It is crucially important to consider such marital partner dynamics when counseling women about HIV and designing HIV prevention and treatment programs.

**Limitations:** while our study relied on nationally representative data, allowing for broader generalizability to married women of reproductive age in Mozambique, it is essential to acknowledge the limitations imposed by the use of secondary data. These limitations include the inability to control for potential biases in the data collection process and the inability to explore additional HIV risk factors that were not encompassed by the IMASIDA survey. Future research endeavors should aim to address these limitations by incorporating more comprehensive data collection strategies to further enhance our understanding of HIV-related risk factors and dynamics within this population.

## Conclusion

Married women or in stable relationships contribute to the prevalence of HIV in Mozambique although in a low proportion. The factors such as age, educational level, number of marriages throughout life, husband/partner circumcised, and total lifetime number of sex partners were found to be significantly associated with HIV seropositivity among married women in Mozambique. These findings underscore the importance of developing HIV/AIDS prevention policies that consider individual, social, and cultural influences on the HIV epidemic and target higher-risk subpopulations of married women such as the implementation of the “Know Your HIV Status” campaign, aimed at the population of men and young people, aiming to improve access to HIV diagnosis, increase combined prevention efforts using all available evidence-based methods to further reduce new HIV infections and other infections; make efforts to improve the supply chain, availability and acceptability of male and female condoms and lubricants. Additionally, strategies to delay the onset of sexual activity, particularly among girls, should be reinforced, including efforts to increase school access, provide socioeconomic support for young people, and raise awareness within families to reduce incidents of domestic violence.

### What is known about this topic

- *This population group (married women) represents one of the highest risk groups for HIV, additionally considering the aspect of polygamy in Africa;*
- *HIV/AIDS surveys in nationally representative populations are essential to assess the quality of the HIV/AIDS program at the national level and report on associated factors*.

### What this study adds

- *HIV/AIDS surveys are necessary as they help to inform about the behavior of the HIV/AIDS virus in specific groups as well as the factors associated with the prevalence of the HIV/AIDS virus in the same population group;*
- *This study highlights the greater importance of designing response policies to combat HIV/AIDS in specific groups*.
